# Spatial Analysis on Dengue Fever Vulnerability in the Provinces of South Sulawesi and East Nusa Tenggara in Indonesia

**DOI:** 10.5334/aogh.4915

**Published:** 2025-12-15

**Authors:** Budi Haryanto, Febi Dwirahmadi, Triarko Nurlambang, Al Asyary

**Affiliations:** 1 Department of Environmental Health, Faculty of Public Health, Universitas Indonesia, Depok – Jawa Barat, Indonesia; 2 Research Center for Climate Change, I‑SER, Universitas Indonesia, Depok – Jawa Barat, Indonesia; 3 Center for Environment and Population Health, School of Medicine, Griffith University, Nathan ‑ Queensland, Australia; 4 Department of Geography, Faculty of Mathematics and Basic Sciences, Universitas Indonesia, Depok – Jawa Barat, Indonesia

**Keywords:** dengue fever control, vulnerability mapping, spatial multi‑criteria evaluation (SMCE)

## Abstract

*Background:* Climate change plays a significant role in increasing dengue fever incidence by altering the habitat suitability for *Aedes* mosquitoes, the primary vectors. The incidence rate of dengue fever in Indonesia is increasing at an alarming rate, and strengthening the surveillance and control of the disease is important to prevent and reduce the risk of infection. This research aims to map and produce vulnerability areas that are suitable for dengue fever vectors by identifying their habitat using environmental and socio‑economic variables.

*Method:* We use six variables as proxy for environmental and socio‑economic drivers of dengue fever, namely 1) distance to pond, 2) distance to drain, 3) building density, 4) distance to health facilities, 5) distance to social activity center, and 6) elevation to represent local temperature and variables are used within the landscape level of research area. This research was conducted in six different regions within two provinces in Indonesia, supported by the incidence rate in each region. Spatial multi‑criteria evaluation (SMCE) was used to map vulnerability areas of dengue fever vector habitats and assign weights and scores to variables according to expert judgements and existing literature.

*Results:* Our findings show that high‑risk areas are located near major water bodies and drainage, lack supporting medical facilities, and are prone to changing climatic conditions. Given the importance of the administrative unit in conducting intervention policies, the calculated total areas of high‑vulnerable zones were given in the research and showed a variation of patterns according to their respective location.

*Conclusion:* Our research suggests that vulnerability areas mapping of dengue fever is needed to control the disease in Indonesia. Thus, this research serves as proof of concept for national‑level mapping.

## Background

The World Health Organization revealed a rapid increase in dengue fever (DF) cases, which have risen 30‑fold over the past 50 years [1]. Considered the most transmissible disease worldwide, the DF virus could potentially infect more than half of the global population. Annual reports show that the number of patients hospitalized with DF ranges from half a million to a million [1]. Climate change is intensifying these environmental conditions, creating more favorable habitats for vector proliferation and virus transmission. Rising temperatures shorten the mosquito’s life cycle and the virus’s incubation period, while erratic rainfall and extended wet seasons lead to more stagnant water sources ideal for mosquito breeding. Consequently, regions previously considered low‑risk are now experiencing endemic transmission, especially in highland areas and urban zones with poor water and waste management [2].

DF remains a significant public health concern in Indonesia, with endemic transmission reported across all provinces. In 2023, Indonesia recorded over 143,000 cases nationally, with a rising trend in eastern regions [3]. South Sulawesi (SS) consistently ranks among the provinces with higher dengue incidence, driven by rapid urbanization, dense population centers such as Makassar, and favorable environmental conditions for *Aedes aegypti* proliferation [3]. In contrast, East Nusa Tenggara (ENT) historically reported lower case numbers, but recent years have shown increasing incidence, particularly in Kupang and Sikka, likely due to changing climate patterns, urban expansion, and limited vector control infrastructure [3]. Compared to national trends, SS reflects a more urban‑driven dengue dynamic, while ENT demonstrates vulnerability due to under‑resourced public health systems and ecological shifts. Both provinces require tailored interventions aligned with local risk profiles to prevent future outbreaks.

Creating a spatial model of DF risk is challenging due to many interrelated factors that could affect dengue. Therefore, it is crucial to understand how these critical factors interact and to create reliable predictive models that can be used to mitigate and control the spread of dengue. This research aims to map and produce vulnerability areas that are suitable for DF vectors by identifying their habitat using environmental and socio‑economic variables.

## Methods

This study applied a spatial multi‑criteria evaluation (SMCE) framework integrated with Geographic Information System (GIS) analysis to identify DF vulnerability zones across six selected cities/regencies in SS and ENT provinces, Indonesia: Makassar, Maros, Luwu Utara (all in SS), and Kupang, Sikka, and Alor (all in ENT). The methodological approach was informed by previous research on dengue risk mapping and adapted to data availability at the city/regency administrative level [4, 5, 6].

Six spatial variables were selected based on their relevance to dengue vector ecology and transmission dynamics, as outlined in Table 1. These variables include: (1) Distance to Pond, representing proximity to standing water bodies that serve as potential breeding sites for *Aedes* mosquitoes [5]; (2) Distance to Drain, acknowledging urban drainage systems as common larval habitats [6]; (3) Building Density, which reflects landscape typology influencing human–vector interactions [7]; (4) Distance to Health Facility, serving as a proxy for both healthcare accessibility and community engagement in dengue prevention [3, 8]; (5) Distance to Social Activity Centers, capturing population movement patterns and urban aggregation [3]; and (6) Elevation, which correlates with temperature, a key factor in the mosquito life cycle and virus development [9–12] (Table 1).

**Table 1:** Six variables used to map the vulnerability area.

NO.	VARIABLE	WEIGHT	CLASS	SCORE
1	Distance to Pond	25	<30 m	3
			30–100 m	2
			>100 m	1
2	Distance to Drain	35	<40 m	3
			40–80 m	2
			>80 m	1
3	Building Density	25	<0.33	1
			0.33–0.66	2
			>0.66	3
4	Distance to Health Facility	5	<500 m	1
			500–1000 m	2
			>1000 m	3
5	Distance to Social Activity Center (market, shopping center, business districts)	5	<500 m	3
			500–1000 m	2
			>1000 m	1
6	Elevation	5	<25	3
			25–100	2
			>100	1

Each criterion was standardized, reclassified, and weighted using expert judgment from epidemiologists and supported by a literature review. A weighted overlay analysis was then performed to generate a composite vulnerability surface, highlighting areas with higher suitability for dengue vector habitats and transmission risk. The final vulnerability map provides a decision‑support tool for local governments in targeting vector control and public health interventions.

## Results

We discuss our results and findings by dividing them into three parts. First, we give clarity on the landscape characteristics of each of our research sites, proxied by our six main variables as shown in the table above. Second, we then further show and describe the results of the GIS analysis using multi‑criteria evaluation (MCE) along with the outbreaks figure or number of cases/occurrences of DF in the respective location in recent years (2018–2020 and 2008–2020), as to show the relation between the two. Lastly, we estimate the total area of high vulnerability spots at the village (*desa*) administrative unit, as we consider this unit as the lowest level of the decision‑making process.

### 1. Makassar city

**Figure 1:**
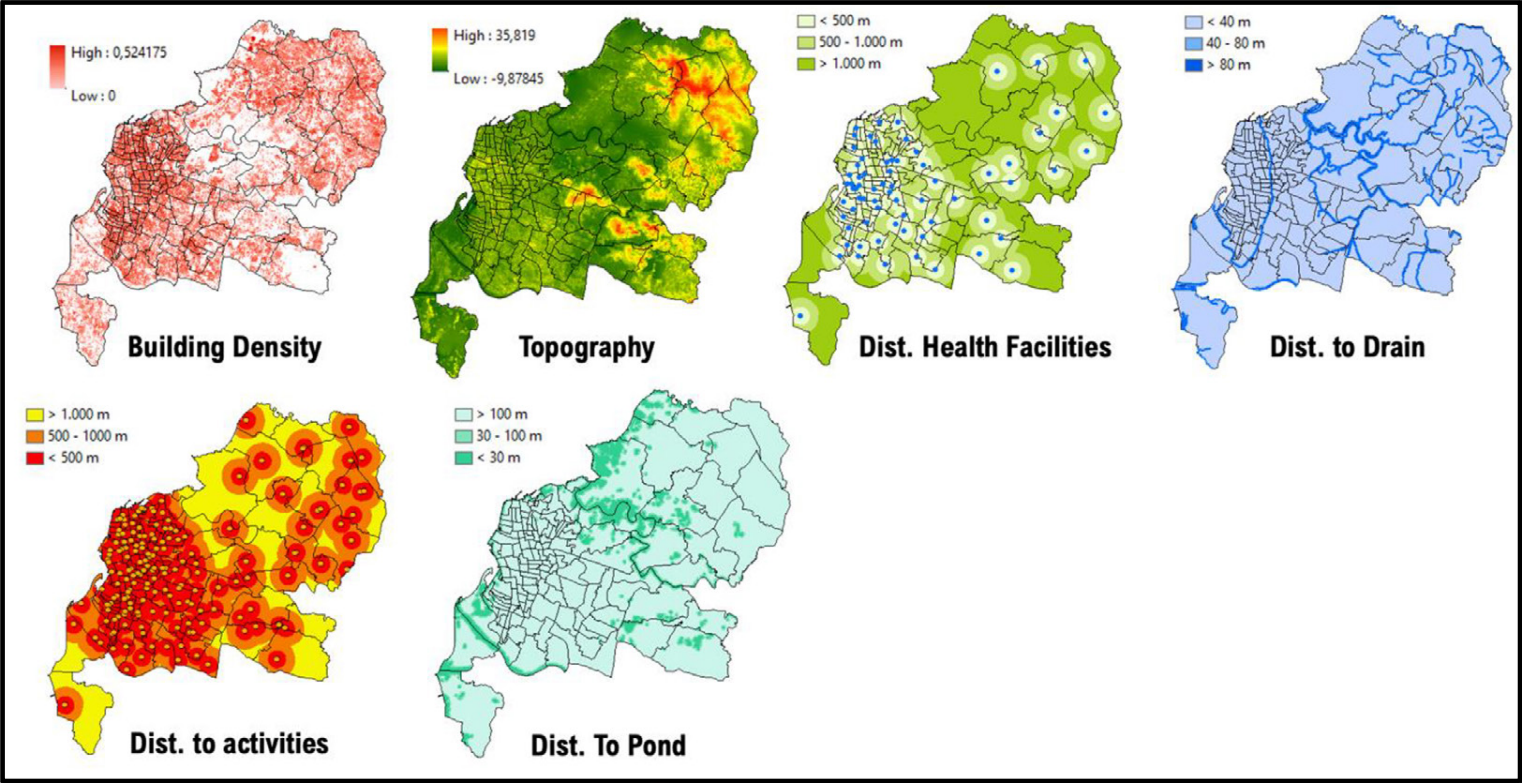
Mapped variables in Makassar City.

**Figure 2:**
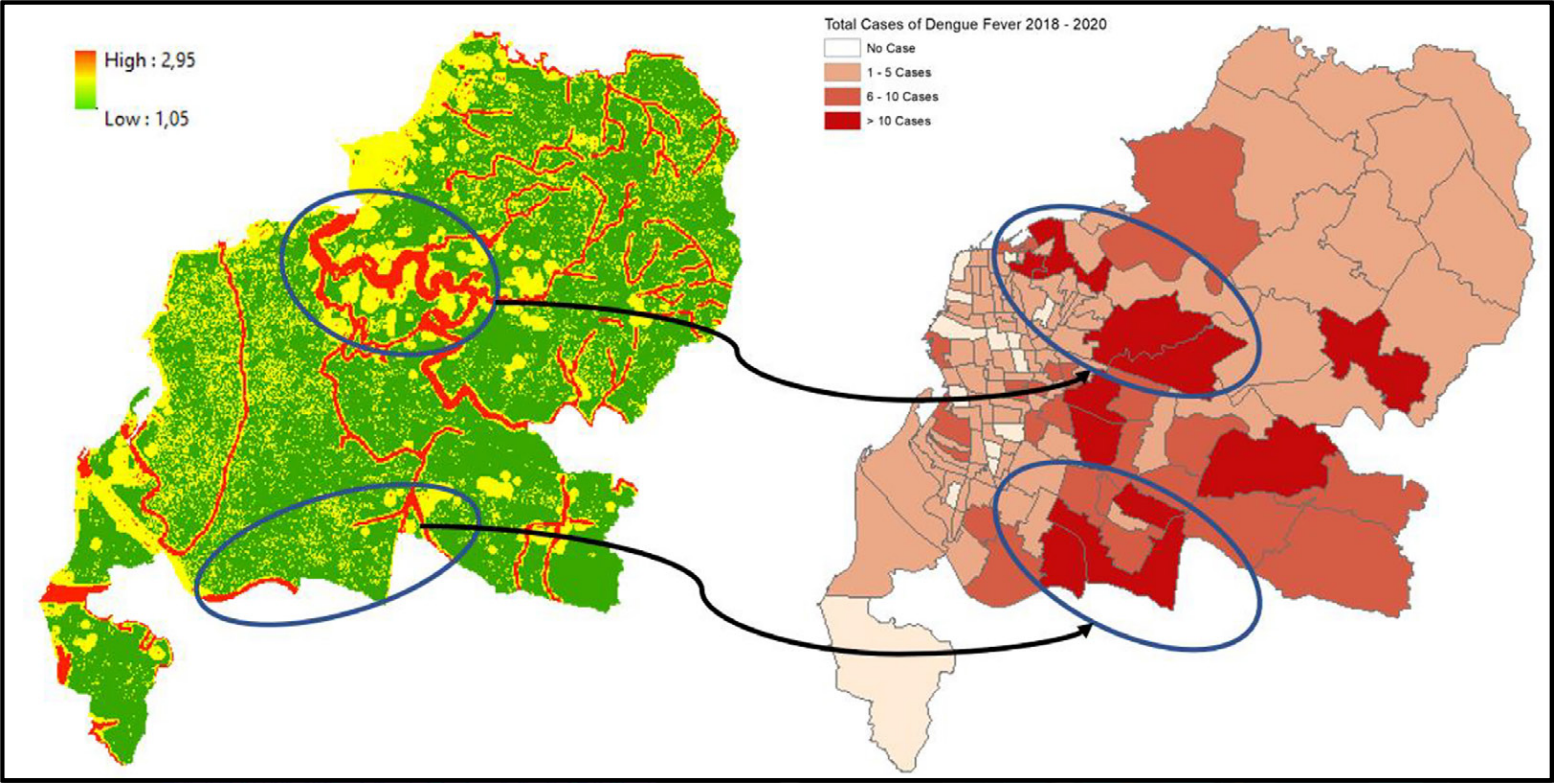
Vulnerability area and outbreaks map of Makassar City.

Data from 2018 to 2020 on outbreak/number of cases of DF within Makassar City shows the highest occurrence, numbering more than 10 cases per region located in the central and south parts of Makassar City, such as Panakkukang, Tamalanrea, Manggala, and Rappocini (Figure 1). In line with our findings, the vulnerability area is located in the center of Makassar City, which is situated within the high‑risk transmission/habitat area of DF vectors (Figure 2).

Villages (*desa*) such as Panaikang, Pampang, and Parang Loe are within the highly vulnerable area of transmission/habitat area. These mentioned areas are mostly situated near permanent water bodies, moderate to high building density, and near the drainage network. Viewing the lowest administrative unit, 15 top villages situated in a high‑risk area are shown on the figure above. The top two of them are Parang Loe and Lakkang, which have the largest high‑risk area of DF transmission/habitat suitability reaching up to 1,379,797 m^2^. These two villages are mainly located in the central part of Makassar City, which is consistent with the recorded data from 2018 to 2020.

### 2. Maros regency

**Figure 3:**
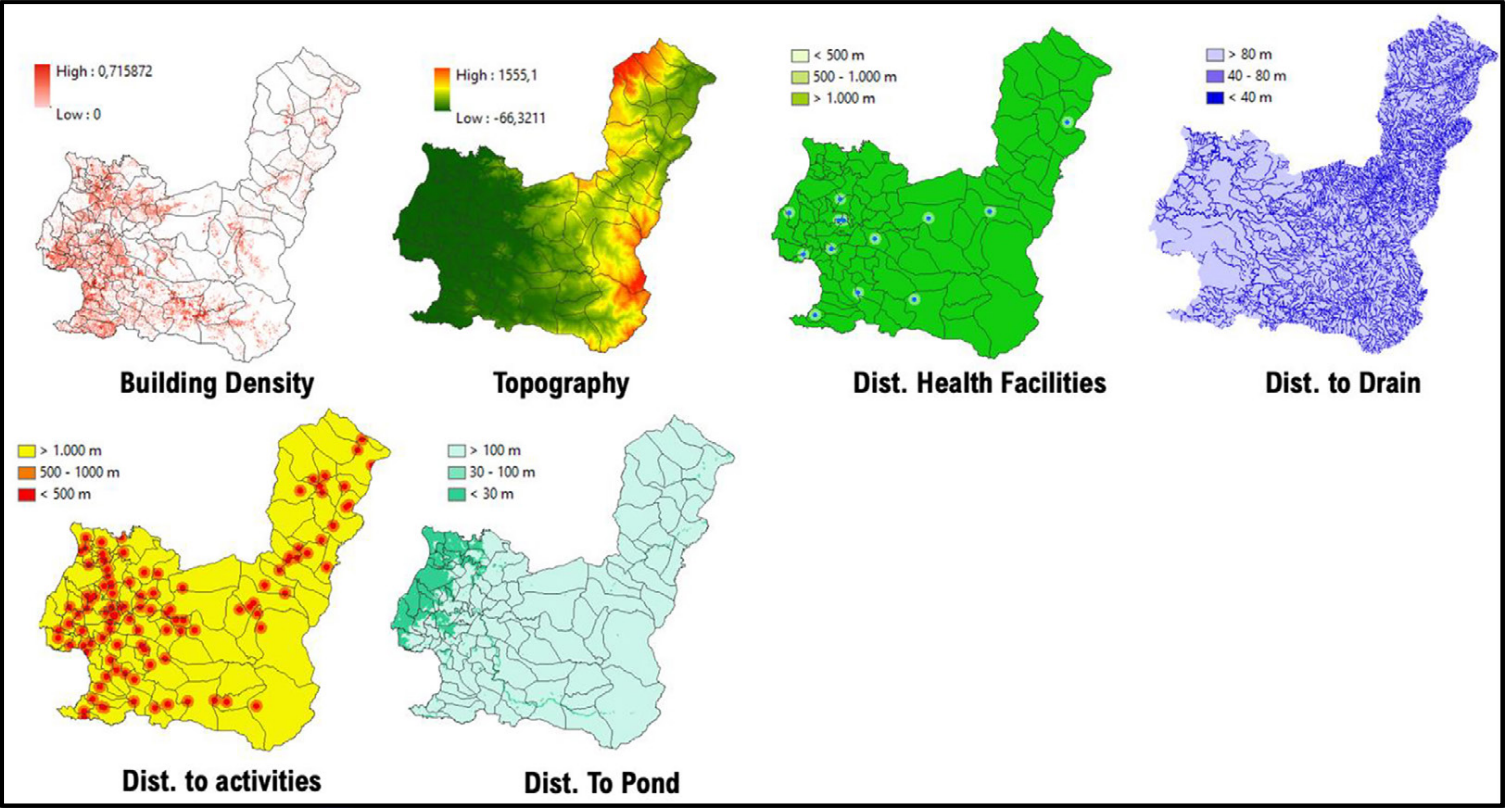
Mapped variables in Maros Regency.

**Figure 4:**
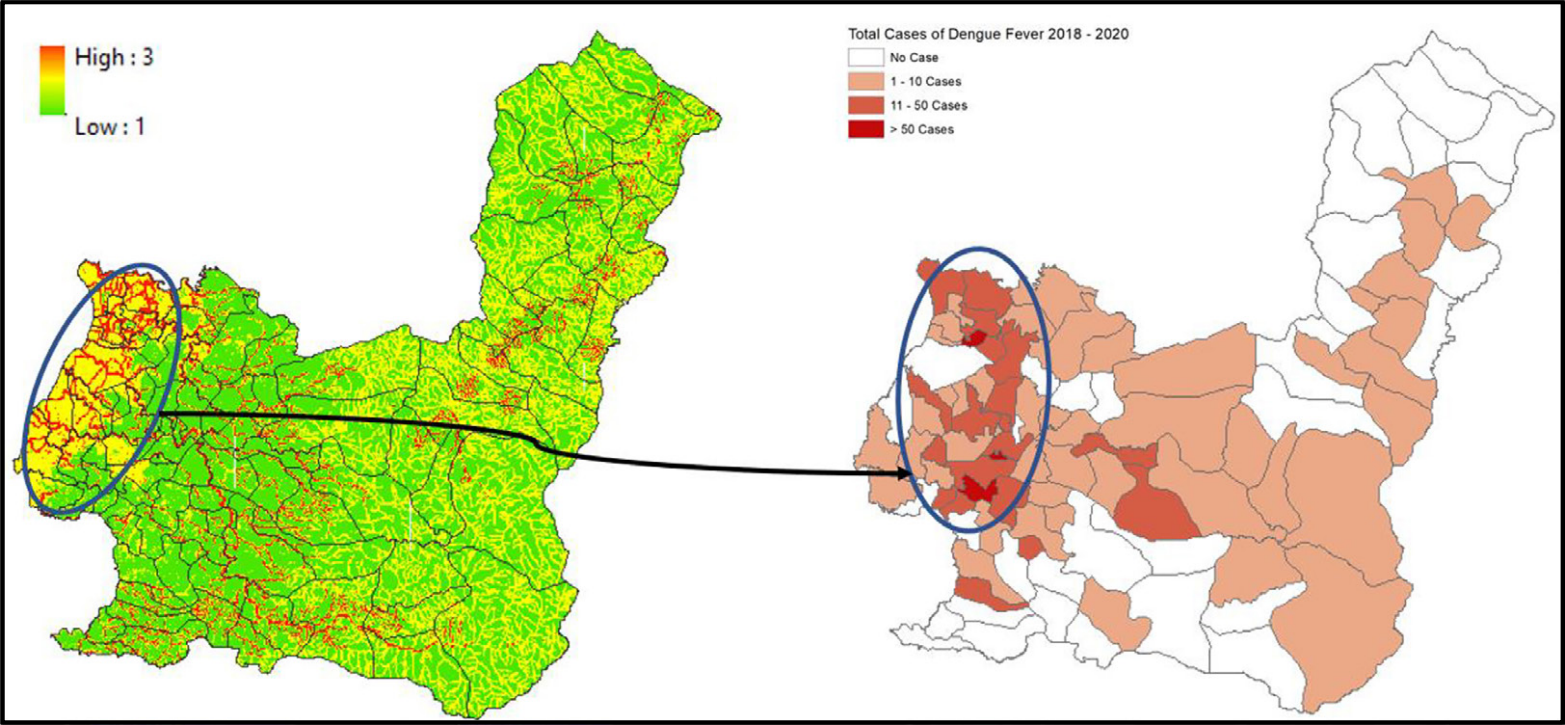
Vulnerability area and outbreaks map of Maros Regency.

Data from 2018 to 2020 on outbreak/number of cases of DF within Maros Regency shows the highest occurrence, numbering more than 50 cases per region located in the western part of Maros Regency, such as Maros, Maros Baru, Lau, and Bontoa village (Figure 3). Linear with our findings, the mapped vulnerability area shows the western parts of Maros Regency situated within a high‑risk transmission/habitat area of DF vectors. Villages (*desa*) such as Maros, Marannu, and Bontoa Maranu are within the highly vulnerable area of transmission/habitat area (Figure 4). These mentioned areas are mostly situated near permanent water bodies, moderate to high building density, and have far‑reaching health facilities. Viewing the lowest administrative unit, 15 top villages that are situated in a high‑risk area are shown on the figure above. The top two of them are Marrannu and Minasa Upa, which have the largest high‑risk area of DF transmission/habitat suitability, reaching up to 4,554,800 m^2^. These two villages are mainly located in the eastern part of Maros Regency, which is consistent with the recorded data from 2018 to 2020.

### 3. Luwu utara regency

**Figure 5:**
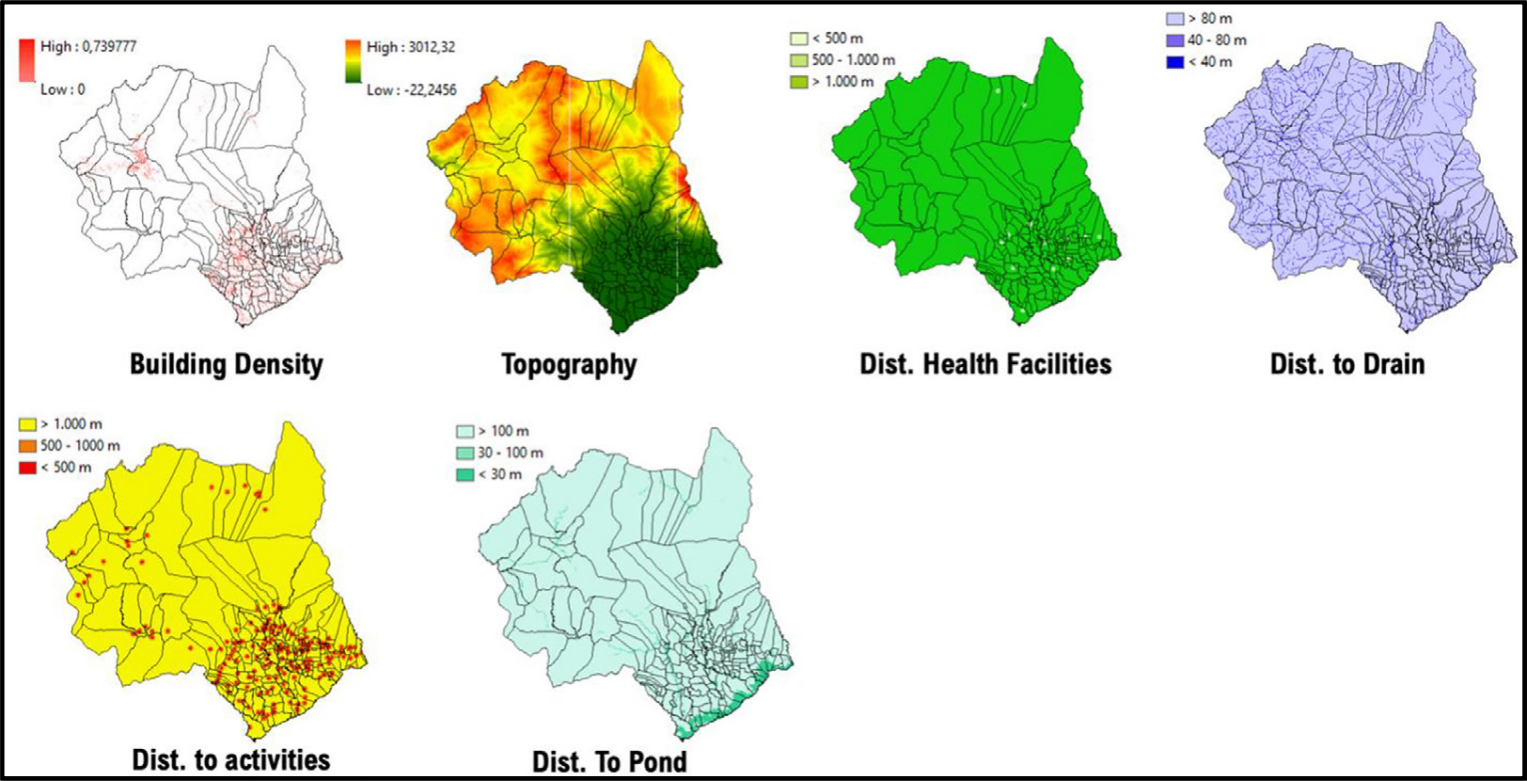
Mapped variables in Luwu Utara Regency.

**Figure 6:**
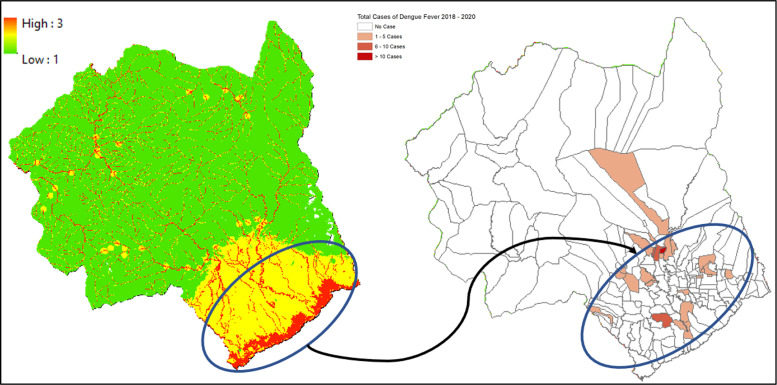
Vulnerability area and outbreaks map of Luwu Utara Regency.

Data from 2018 to 2020 on outbreak/number of cases of DF within Luwu Utara Regency shows the highest occurrence, numbering more than 10 cases per region located in the southeast part of Luwu Utara stretching along the coastline, such as suburban Malangke, Malangke Barat, and Bone‑Bone (Figure 5). Consistent with our findings, the mapped vulnerability area shows southeast parts of Luwu Utara city are situated within a high risk of transmission/habitat area of DF vectors (Figure 5). Villages (*desa*) such as suburban Malangke, Lebone, and Pao are within the highly vulnerable area of transmission/habitat area. These mentioned areas are mostly situated near the coastline and permanent water bodies, with moderate to low building density, and low medical facilities (Figure 6).

Viewing the lowest administrative unit, 15 top villages that are situated in a high‑risk area are shown on the figure above. The top two of them are Marante and Leboni, which have the largest high‑risk area of DF transmission/habitat suitability, reaching up to 26,688,011 m^2^. These two villages are separately located in the northwest part and southeast of Luwu Utara. Points for consideration that Marante village originally had larger administrative areas with major water bodies flowing, and low access to medical facilities.

### 4. Kupang city

**Figure 7:**
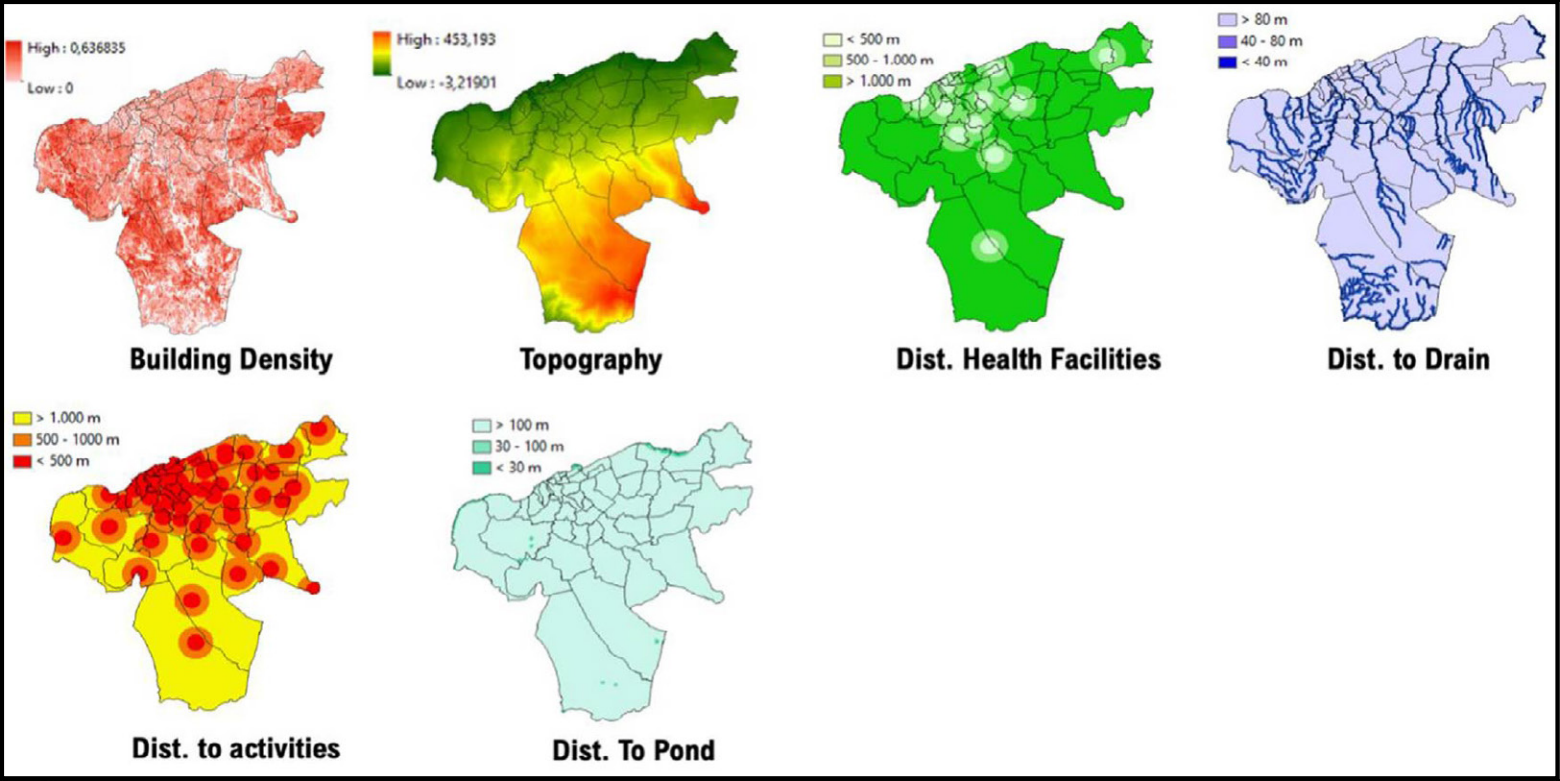
Mapped variables in Kupang City.

**Figure 8:**
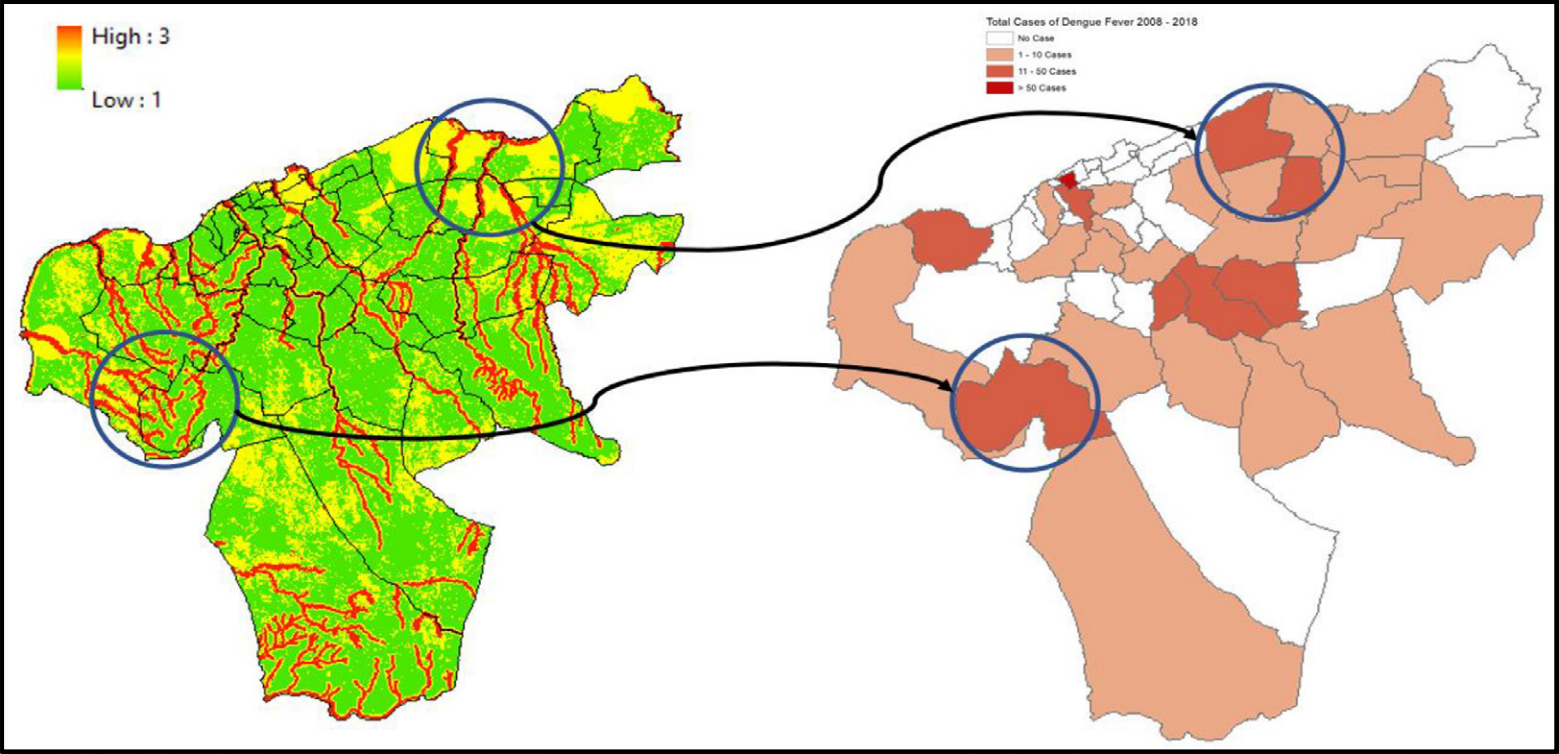
Vulnerability area and outbreaks map of Kupang City.

Data from 2008 to 2020 on outbreak/number of cases of DF within Kupang City shows the highest occurrence, numbering more than 50 cases per region located in the north and west parts of Kupang City**,** such as Kelapa Lima, and the Alak region (Figure 7). Consistent with our findings, the mapped vulnerability area shows that most parts of Kupang city are situated within a high risk of transmission/habitat area of DF vectors. Villages (*desa*) such as Naioni, Alak, and Penkase‑Oeleta are within the highly vulnerable area of transmission/habitat area (Figure 8). These mentioned areas are mostly situated near permanent water bodies and near drainage/major river networks. Viewing the lowest administrative unit, 15 top villages that are situated in a high‑risk area are shown on the figure above. The top two of them are Naioni and Alak, which have the largest high‑risk area of DF transmission/habitat suitability, reaching up to 3,920,300 m^2^. These two villages are mainly located in the central and northern parts of Kupang City, which is consistent with the recorded data from 2018 to 2020.

### 5. Sikka regency

**Figure 9:**
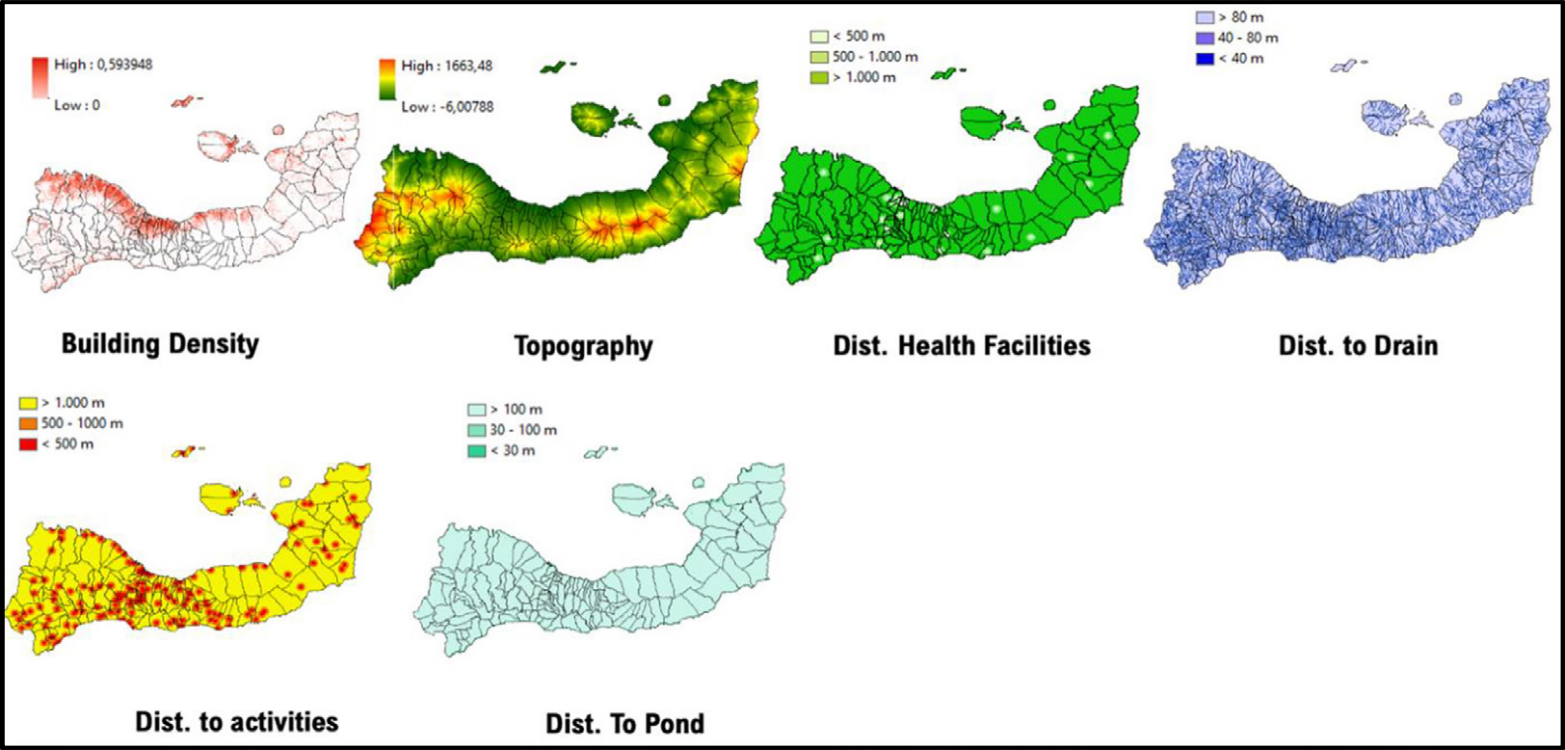
Mapped variables in Sikka Regency.

**Figure 10:**
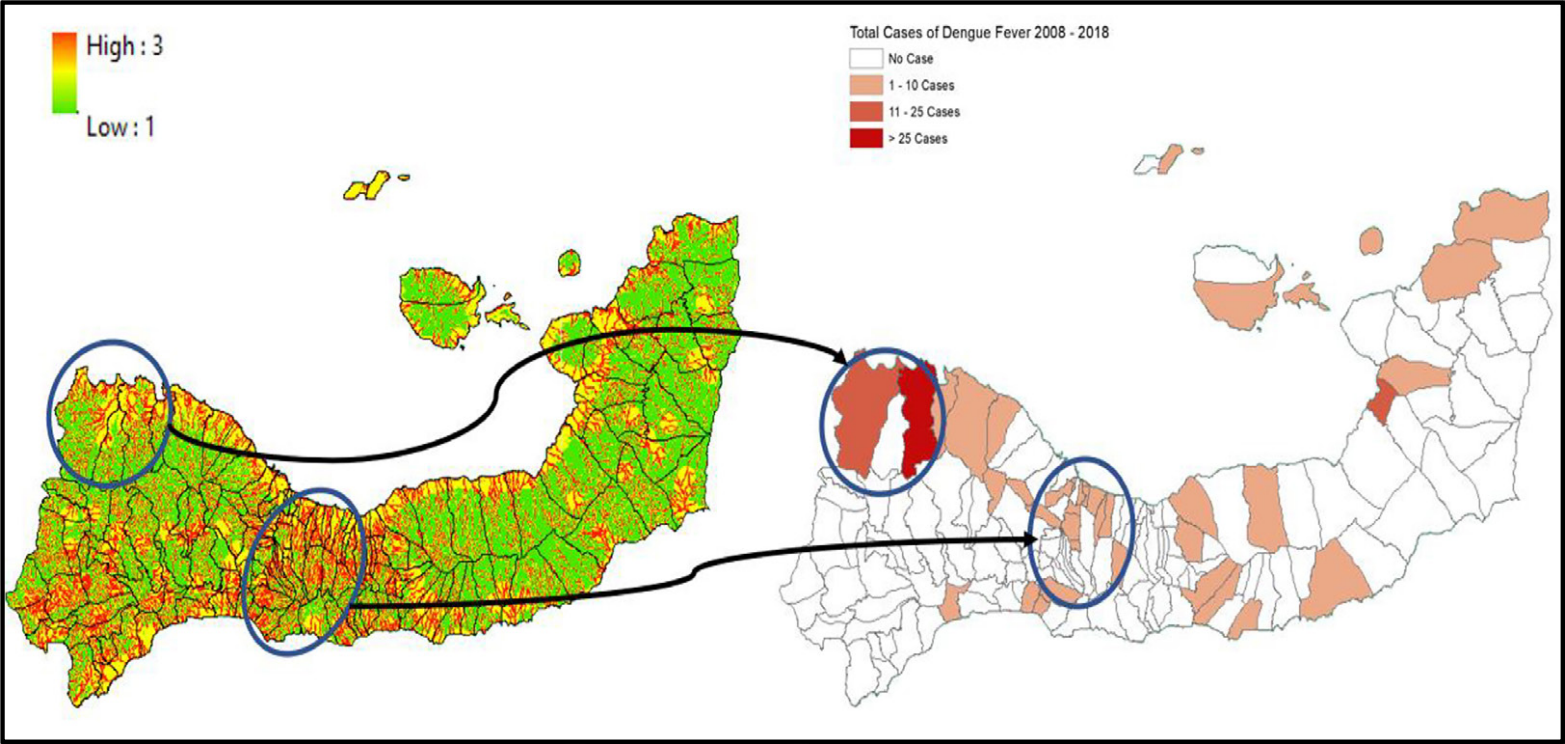
Vulnerability area and outbreaks map in Sikka Regency.

Data from 2008 to 2020 on outbreak/number of cases of DF within Sikka Regency shows the highest occurrence, numbering more than 25 cases per region located in the western part of Sikka Regency, such as Magepanda, and Alok Barat (Figure 9). Linear with our findings, the mapped vulnerability area also shows the western parts of Sikka Regency situated within a high‑risk transmission/habitat area of DF vectors (Figure 10). Villages (*desa*) such as Reroroja and Magepanda are within the highly vulnerable area of transmission/habitat area. These mentioned areas are mostly situated within a dense urban landscape, and further away from medical facilities. Viewing the lowest administrative unit, 15 top villages that are situated in a high‑risk area are shown on the figure above. The top two of them are Reroroja and Magepanda, which have the largest high‑risk area of DF transmission/habitat suitability, reaching up to 14,266,900 m^2^. These two villages are mainly located in the western part of Sikka Regency, which is consistent with the recorded data from 2018 to 2020.

## Alor Regency

**Figure 11:**
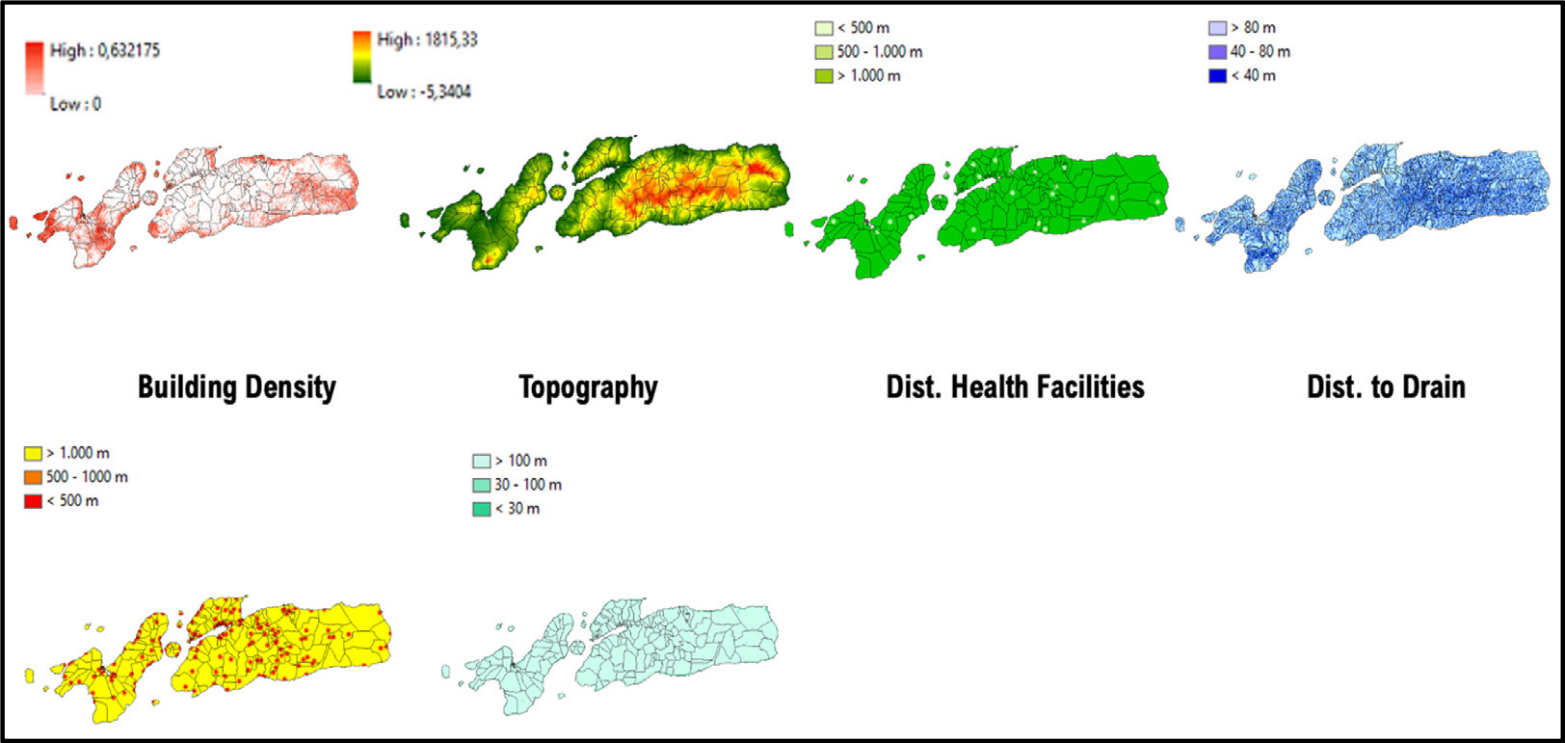
Mapped variables in Alor Regency.

**Figure 12:**
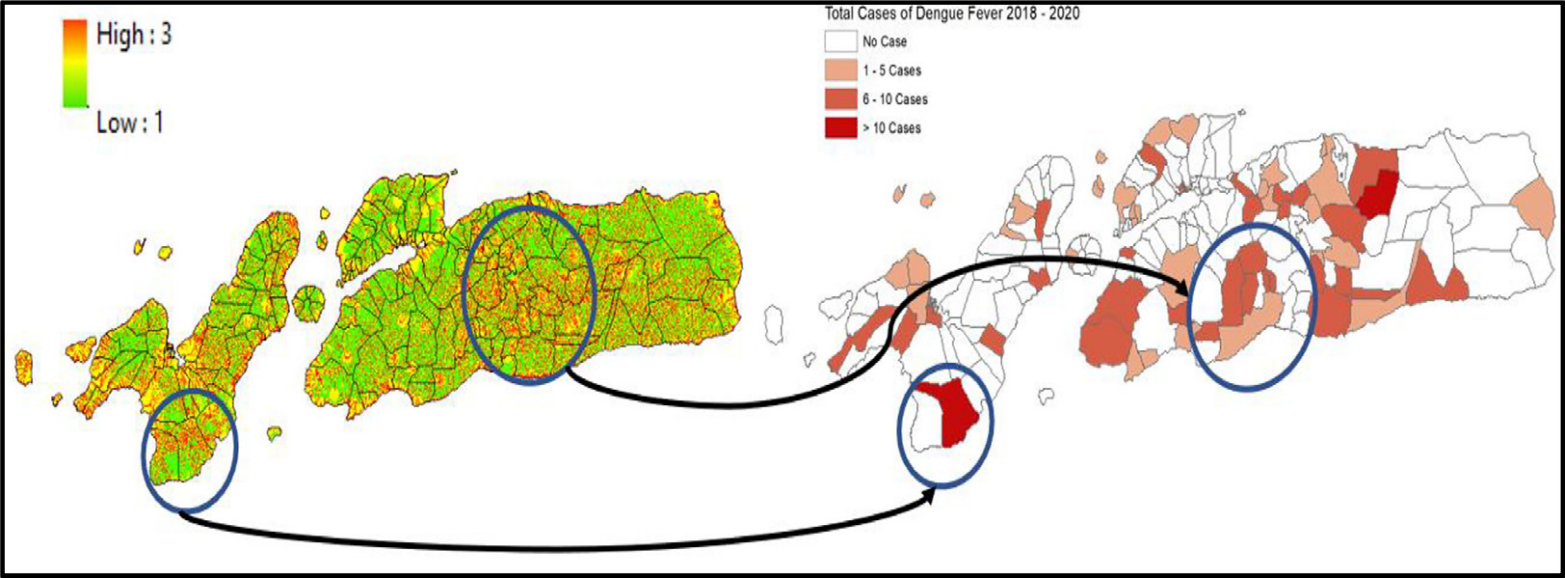
Vulnerability area and outbreaks map of Alor Regency.

Data from 2008 to 2020 on outbreak/number of cases of DF within Alor Regency shows the highest occurrence, numbering more than 10 cases per region located in the central and western parts of Alor, such as Pantar Tengah and Alor Barat Daya (Figure 11). Consistent with our findings, the mapped vulnerability area shows that the central and western parts of Alor Regency are situated within a high‑risk transmission/habitat area of DF vectors (Figure 12). Villages (*desa*) such as Panaikang, Pampang, and Parang Loe are within the highly vulnerable area of transmission/habitat area. These mentioned areas are mostly situated near permanent water bodies, moderate to high building density, and near the drainage network.

## Discussion

Climate change plays a significant role in increasing DF incidence by altering the habitat suitability for *Aedes* mosquitoes, the primary vectors. Rising temperatures accelerate mosquito development and viral replication, while changes in precipitation patterns expand breeding sites, particularly in tropical regions like Indonesia. Warmer climates also shift the geographical range of transmission to higher altitudes and previously unaffected areas. These environmental changes, combined with urbanization and population growth, enhance transmission dynamics and complicate control efforts [13, 14].

This study employed an SMCE integrated with GIS to identify DF vulnerability zones across six cities and regencies in South Sulawesi (Table 2) and ENT (Table 3) Indonesia. By combining environmental and socio‑economic variables—such as proximity to ponds and drains, building density, distance to health facilities and social activity centers, and elevation—our research offers a comprehensive spatial risk model aligned with historical outbreak data. This methodology proves useful for enhancing local disease surveillance and informing targeted interventions.

**Table 2:** MCE results of Makassar City, Maros Regency, and Luwu Utara Regency.

VARIABLES	MAKASSAR CITY	MAROS REGENCY	LUWU UTARA REGENCY
Building density	Ujung Pandang subdistrict is the most crowded region, where covered by dense buildings and settlements	Maros, Maros Baru, Marusu, and Bontoa are the most crowded regions where covered with dense buildings and settlements	The most crowded areas are in the southeast (suburban Malangke and Malangke Barat), with moderate to low buildings and settlements
Topography	The highest elevation is located in the east and northeast (suburban Biring Kanaya and Tamalanrea, 30 MASL)	The highest elevation is located in the east and northeast (suburban Malawa and Cenrana, 1555 MASL)	The highest elevation is located in the west and northwest (suburban Seko and Rampi, 3012 MASL)
Distance to health facilities	The west region is better supported with medical facilities	All are located more than 1 km away using Euclidean distance	The east region is more supported with medical facilities than the northwest region
Distance to drainage	The major drain flows from the northeast region to the west	The major drain flows from the northeast region to the west	The major drain flows from the northwest and west region to the east
Distance to the activity hotspot	The majority in the Ujung Pandang Subdistrict and Mamajang	The majority in suburban Maros, Maros Baru, and Marusu	The majority are located in the southeast region (suburban Malangke, Malangke Barat, and Baebunta)
Distance to ponds	It stretched and flew from the east region (suburban Biring Kanaya) to the west to Ujung Pandang	Major and permanent water bodies are located within the suburban Maros, Maros Baru, Bontoa, and Lau regions	Major and permanent water bodies located near the ocean, including suburban Malangke and Malangke Barat

**Table 3:** MCE results of Kupang City, Sikka Regency, and Alor Regency.

VARIABLES	KUPANG CITY	SIKKA REGENCY	ALOR REGENCY
Building density	Kupang City is its most crowded region, where covered by dense buildings and settlements	Magepanda and Alok Barat as its most crowded regions, covered by dense buildings and settlements, and located along the western coast	Alor Timur and Pantar Tengah are the most crowded regions where covered by dense buildings and settlements
Topography	The highest elevation is located in the east and northeast (suburban Biring Kanaya and Tamalanrea, 30 MASL)	The highest elevation is located in the west and central region (Tana Wawo and Mego, 1663 MASL)	The highest elevation is located at the center of the island (Alor Selatan, 1815 MASL)
Distance to health facilities	The west region is better supported with medical facilities	Shown that minimal medical facilities and a health center are located in Sikka Regency	The western regions are better supported with medical facilities
Distance to drainage	The major drain flows from the northeast region to the west	The major drain flows from the northeast region to the west	The major drain flows from the center of the region
Distance to the activity hotspot	The majority in the Ujung Pandang Subdistrict and Mamajang	Crowded region such as Magepanda and Alok Barat shows major activity hotspots compared to the other regions	The majority in the region of Alor Timur and Pantar Tengah
Distance to ponds	It stretched and flew from the east region (suburban Biring Kanaya) to the west to Ujung Pandang	Major and permanent water bodies, such as ponds and major rivers, are on average further than 100 m from Sikka Regency	Major and permanent water bodies generally farther than 100 m

Our results affirm the assertion by Bhatt et al. that dengue risk is spatially heterogeneous and driven by complex ecological and social interactions [15]. In the urbanized province of South Sulawesi, particularly in Makassar and Maros, high building density, proximity to permanent water bodies, and limited access to drainage infrastructure contributed significantly to higher vulnerability levels. Regions such as Panakkukang, Manggala, and Bontoa were identified as hotspots with dengue incidence exceeding 10–50 cases between 2018 and 2020, consistent with previous research linking urban density with *Aedes aegypti* breeding habitats [10].

Luwu Utara, while less urbanized, showed high vulnerability along coastal and low‑lying areas like Malangke and Bone‑Bone. These areas share a combination of risk factors, including poor drainage, limited health infrastructure, and environmental conditions conducive to vector survival. The findings correspond with those of Louis et al. and Khan et al., who emphasize the influence of spatial and environmental variables in shaping disease transmission patterns [16, 17].

In ENT, vulnerability patterns indicate emerging risk linked to urban expansion, changing climatic conditions, and infrastructural limitations. Kupang City, for example, experienced outbreaks of more than 50 cases annually in areas such as Alak and Naioni. These locations are characterized by moderate to high building density, proximity to water bodies, and insufficient drainage systems. Similarly, in Sikka and Alor, rural‑urban transition zones such as Magepanda and Alor Barat Daya are vulnerable due to environmental degradation and sparse medical services. These findings echo the warnings of Ebi and Nealon and Seah et al. on how climate change and elevation, which influence local temperature, can intensify vector breeding cycles and geographic distribution [14, 18].

The spatial concordance between vulnerability maps and historical dengue cases across study sites validates the model’s predictive utility. SMCE allows for a nuanced prioritization of intervention areas. For instance, the village‑level (*desa*) estimates of high‑risk zones provide actionable insight for local health authorities. This approach supports the WHO’s (2022) recommendation for strengthening sub‑national dengue surveillance systems, particularly in countries such as Indonesia, where decentralization plays a crucial role in public health governance.

In Makassar and Maros, top villages such as Parang Loe and Marrannu were identified as having the largest vulnerable areas, up to 1.38 million and 4.55 million square meters, respectively. These findings correlate with proximity to ponds, dense settlements, and limited access to healthcare, reinforcing conclusions by Buxton et al. that vector abundance is closely tied to standing water and thermal fitness [19]. Similarly, in Kupang and Sikka, areas such as Naioni and Magepanda exhibited significant vulnerability zones, aligned with outbreak histories, poor medical access, and physical barriers that hindered service delivery.

Despite the strengths of our approach, certain limitations must be addressed. First, the static nature of our environmental variables does not fully capture seasonal variability or real‑time shifts in vector dynamics. As pointed out by Ewing et al., temperature, rainfall, and humidity—absent in our current model—play vital roles in *Aedes* lifecycle progression [12]. Second, while expert judgment for assigning weights and scores ensures methodological transparency, it may introduce subjectivity that could be refined through participatory methods or machine learning‑based calibration. Incorporating finer‑resolution data and integrating temporal variables in future studies would increase robustness and applicability.

Moreover, this study underlines the need for interdisciplinary collaboration in vector control. Health authorities must work with urban planners, environmental agencies, and climate scientists to build comprehensive, long‑term strategies. For example, environmental design initiatives that improve drainage systems, promote green infrastructure, and regulate urban growth can reduce suitable breeding sites. Our findings are consistent with Marti et al., who suggest integrating remote sensing and earth observation technologies for improved urban landscape monitoring [4].

In terms of public health policy, our study demonstrates that GIS‑based vulnerability mapping should be institutionalized in Indonesia’s dengue prevention programs. The Ministry of Health and local governments can use the mapped outputs to guide fogging operations, resource allocation, and public awareness campaigns. Spatial models also support early warning systems and can inform climate‑resilient health planning, as advocated by Acharya et al. [20].

## Conclusion

This research highlights the spatial heterogeneity and multi‑factorial nature of DF vulnerability in Indonesia. South Sulawesi, with its dense urban centers, faces a different set of challenges compared to ENT, where infrastructural deficits and ecological change drive vulnerability. Nonetheless, both provinces require context‑specific strategies rooted in evidence‑based planning and spatial analytics.

By applying a replicable SMCE‑GIS framework, this study contributes to the growing body of literature on spatial epidemiology and offers a scalable tool for national dengue mapping. Future efforts should incorporate climate forecasting, community‑level data, and participatory risk assessments to further enhance preparedness and response. Given the increasing burden of dengue in Indonesia—especially in the context of climate change and rapid urbanization—adopting a proactive, geographically informed public health strategy is no longer optional but essential.
